# Vitamin D Reprograms Non-Coding RNA Networks to Block Zika Virus in Human Macrophages

**DOI:** 10.3390/pathophysiology33010015

**Published:** 2026-02-03

**Authors:** Julieta M Ramírez-Mejía, Geysson Javier Fernandez, Silvio Urcuqui-Inchima

**Affiliations:** 1 Grupo Biología y Control de Enfermedades Infecciosas, Universidad de Antioquia UdeA, Medellín 050010, Colombia; jmramirez2@eafit.edu.co (J.M.R.-M.); geysson.fernandez@udea.edu.co (G.J.F.); 2 Grupo Inmunovirología, Departamento de Microbiología y Parasitología, Facultad de Medicina, Universidad de Antioquia UdeA, Calle 70 No. 52-21, Medellín 050010, Colombia

**Keywords:** competing endogenous RNA, lncRNA, miRNA, mRNA, pro-inflammatory response, antiviral response, virus, bioinformatics

## Abstract

**Background:** Zika virus (ZIKV), a mosquito-borne flavivirus, is associated with congenital malformations and neuroinflammatory disorders, highlighting the need to identify host factors that shape infection outcomes. Macrophages, key targets and reservoirs of ZIKV, orchestrate both antiviral and inflammatory responses. **Methods:** Vitamin D (VitD) has emerged as a potent immunomodulator that enhances macrophage antimicrobial activity and regulates inflammation. To investigate how VitD shapes macrophage responses to ZIKV, we reanalyzed publicly available RNA-seq and miRNA-seq datasets from monocyte-derived macrophages (MDMs) of four donors, differentiated with or without VitD and subsequently infected with ZIKV. **Results:** Differential expression analysis identified long non-coding RNAs (lncRNAs), microRNAs (miRNAs), and mRNAs integrated into competing endogenous RNA (ceRNA) networks. In VitD-conditioned and ZIKV-infected MDMs, 65 lncRNAs and 23 miRNAs were significantly modulated. Notably, lncRNAs such as HSD11B1-AS1, Lnc-FOSL2, SPIRE-AS1, and PCAT7 were predicted to regulate immune and metabolic genes, including G0S2, FOSL2, PRELID3A, and FBP1. Among the miRNAs, let-7a and miR-494 were downregulated, while miR-146a, miR-708, and miR-378 were upregulated, all of which have been previously implicated in antiviral immunity. Functional enrichment analysis revealed pathways linked to metabolism, stress responses, and cell migration. ceRNA network analysis suggested that SOX2-OT and SLC9A3-AS1 may act as molecular sponges, modulating regulatory axes relevant to immune control and viral response. **Conclusions:** Despite limitations in sample size and experimental validation, this study provides an exploratory map of ncRNA–mRNA networks shaped by VitD during ZIKV infection, highlighting candidate molecules and pathways for further studies on host–virus interactions and VitD-mediated immune regulation.

## 1. Introduction

Zika virus (ZIKV) is a mosquito-borne flavivirus that has garnered global attention due to its association with congenital malformations, particularly microcephaly, and its potential to cause neurological complications, such as Guillain-Barré syndrome, in adults [1,2]. In recent years, substantial progress has been made in elucidating the immune mechanisms engaged during ZIKV infection. Both innate and adaptive responses are crucial, but their dysregulation can lead to immunopathology [3,4]. These insights have supported the development of vaccine candidates, some of which have had clinical evaluations. At the same time, ZIKV has been shown to exploit host pathways, including miRNA regulation, to evade immune surveillance [5]. Given the absence of specific antiviral therapies, a deeper understanding of host immune responses remains essential for guiding the development of effective interventions.

Recent research has highlighted the immunomodulatory effects of Vitamin D (VitD), a fat-soluble steroid hormone, in bolstering macrophage-mediated antiviral responses [6,7]. VitD promotes an antiviral state by upregulating the expression of antimicrobial peptides and proinflammatory cytokines in macrophages [8]. For instance, VitD supplementation has been shown to reduce ZIKV replication in vitro [9]. This dual role, enhancing host immune defense while directly impairing viral replication, underscores the potential of VitD as a key modulator in the host response to ZIKV. Yet, despite this recognized potential, the molecular mechanisms through which VitD shapes antiviral immunity during ZIKV infection remain only partially understood.

MicroRNAs (miRNAs) and long non-coding RNAs (lncRNAs) are emerging as critical regulators of gene expression in diverse biological processes, including antiviral immunity [10,11]. Beyond their individual roles, these ncRNAs participate in intricate regulatory circuits, where miRNAs and lncRNAs interact with mRNA targets to modulate the expression of genes involved in the antiviral defense machinery [5,12] A key example is the competing endogenous RNA (ceRNA) mechanism, in which lncRNAs act as a molecular sponge that sequesters miRNA, thereby relieving repression of specific mRNA and modulating antiviral defense pathways [13,14]. Interestingly, several VitD-modulated genes during ZIKV infection are targets of miRNA and lncRNA [15,16], suggesting the involvement of complex post-transcriptional regulatory mechanisms. However, the contribution of ceRNA interaction to VitD-mediated immune modulation in ZIKV infection remains largely unexplored.

Considering our previous findings [15,16], this study aims to investigate how VitD shapes lncRNA-mRNA interaction in ZIKV-infected macrophages. To this end, we reanalyzed RNA-Seq and miRNA-Seq datasets from monocyte-derived macrophages (MDMs) differentiated with or without VitD before ZIKV infection. Differentially expressed lncRNAs and miRNAs were integrated with mRNA targets to construct a ceRNA regulatory network. This genome-wide approach provides novel insights into the molecular pathway and regulatory circuits through which VitD may modulate host response to ZIKV.

## 2. Materials and Methods

### 2.1. Ethics Statement

This study was conducted with the approval of the Ethics Committee at the “Sede de Investigación Universitaria-Universidad de Antioquia”, Medellín, Colombia (approval code: 16-08-702; 28 April 2016). These ethical approvals are in accordance with the Declaration of Helsinki. All patients in this study provided written informed consent before blood collection.

### 2.2. Culturing of Human Monocytes and Differentiation into Monocyte-Derived Macrophages

Peripheral blood was obtained from four healthy donors and collected in tubes containing 4% *v*/*v* EDTA. Peripheral blood mononuclear cells (PBMCs) were isolated by gradient centrifugation using Ficoll-Histopaque (Sigma-Aldrich, St. Louis, MO, USA) at 650× *g* for 30 min. Platelet depletion was achieved through three washes with 1× PBS (Sigma-Aldrich) at 250× *g* for 10 min. The percentage of CD14+ cells was determined by flow cytometry, with 5 × 10^5^ cells stained with 1 μL of anti-CD14 (eBiosciences, San Diego, CA, USA) for 30 min. Monocytes were isolated from total PBMCs using a plastic adherence procedure. Non-adherent cells were removed by washing twice with 1× PBS, after which monocyte-derived macrophages (MDMs) were differentiated either in the absence (MDM) or the presence of 1 nM 1,25-dihydroxyvitamin D3 (D3-MDMs; Sigma-Aldrich). Cells were cultured for 6 days in RPMI 1640 medium supplemented with 10% autologous serum and maintained at 37 °C with 5% CO_2_, with the medium replaced every 24 h until the completion of differentiation. The control group (MDMs) was treated with vehicle [0.1% ethanol (EtOH)]. This methodology follows a previously published protocol [17] in which differentiation into macrophages was confirmed by morphology and surface marker expression. All experiments were performed using cells from four independent healthy donors (*n* = 4), yielding four biological replicates per experimental condition. Each donor’s cells were processed independently through all experimental steps (differentiation, treatment, infection, and RNA extraction).

### 2.3. ZIKV Infection and Treatments

On day 6 of culture, MDMs and D3-MDMs were infected by ZIKV strain COL345Si-Asian (kindly gifted by Professor Blanco P. Universidad de Sucre, Colombia) at a multiplicity of infection (MOI) of 5 (ZIKV-MDM and ZIKV-D3-MDM, respectively) in serum-free RPMI-1640. The cells were incubated for 2 h at 37 °C with 5% CO_2_. Subsequently, the supernatant was removed, and the cells were washed with 1× PBS. The medium was then replenished with RPMI containing 10% FBS and cultured at 37 °C with 5% CO_2_. Harvesting of MDMs was performed at 24 h post-infection (hpi).

### 2.4. RNA Isolation, Library Preparation, and miRNA-Seq

Total RNA was extracted using the Direct-zol RNA MiniPrep kit (Zymo Research, Irvine, CA, USA) following the manufacturer’s instructions. RNA integrity was evaluated by assessing the ratio of 28S and 18S rRNA on a 1% agarose gel. For miRNA sequencing, libraries were prepared from two infected groups (ZIKV-MDM and ZIKV-D3-MDM) and two uninfected controls (MDM and D3-MDM) using a single-adapter ligation and circularization strategy [18]. Libraries were sequenced as 50 bp single-end reads, yielding depths of 7 to 12 million reads per sample.

### 2.5. Read Alignment and Differential miRNA Expression Analysis

FASTQ files from the miRNA libraries were quality-filtered with a Phred score cutoff of ≥30, and Illumina adapter sequences were trimmed using Trimmomatic (V0.32). Single-end reads were aligned to miRBase v22 with Bowtie using the parameters -n 0 -l 8 -a --best --strata --phred33-quals. miRNA counts were generated using FeatureCounts (default settings) and normalized with DESeq2 (version 1.32). Differential expression was defined as an absolute fold change (|fold change| ≥ 2 and a false discovery rate (FDR) ≤ 0.05. Curated miRNA annotations were obtained from MirGeneDB.

### 2.6. Read Alignment and Differential lncRNA Expression Analysis

RNA-seq data from ZIKV-MDMs and ZIKV-D3-MDMs and their respective controls (MDM and D3-MDM) are publicly available in the NCBI GEO database under accession number GSE209698. Each experimental group consisted of four biological replicates (*n* = 4 independent donors). Data were reanalyzed by mapping to the human reference genome (hg38) using STAR, and differential expression was assessed with DESeq2 under a negative binomial distribution model. Expression profiles were obtained for mRNAs (RefSeq databases) and lncRNAs (NONCODE database). Differentially expressed lncRNAs were defined as those with an absolute |fold change| ≥ 2 and FDR ≤ 0.05.

### 2.7. mRNA Differential Expression

For mRNA expression analysis, we used publicly available data from the Gene Expression Omnibus (GEO; accession number GSE209698). Differential expression was assessed following the methodology outlined in [19]. Briefly, we performed differential gene expression analysis using DESeq2, incorporating donor index as a covariate in the design formula to control for inter-individual genetic background differences. To minimize false-positive discoveries, we applied stringent statistical thresholds, considering genes differentially expressed (DEGs) only when they met both criteria: false discovery rate (FDR) ≤ 0.05 and an absolute log2 fold change > 1. This conservative approach ensured robust identification of biologically meaningful expression changes while accounting for donor-specific variability.

### 2.8. VDR Motif Analysis

We investigated VDR (Vitamin D Receptor) binding to the promoters of differentially expressed genes in the THP-1 macrophage cell line. THP-1 cells were stimulated with 1,25-dihydroxyvitamin D3 (1,25(OH)2D3) to activate VDR-mediated signaling, and ChIP-Seq data (GEO; accession number GSE2096981) were used to map VDR binding sites. Visualization with the Integrated Genome Viewer (IGV) version 2.3.90 enabled the identification of potential VDR occupancy within the target gene promoters.

### 2.9. Gene Enrichment Analysis

Gene enrichment analysis was performed using EnrichR, with the Kyoto Encyclopedia of Genes and Genomes (KEGG) pathways and Gene Ontology (GO) Biological Process database. Enrichment results were expressed as the percentage of genes per term, calculated by EnrichR as overlap. KEGG and GO terms were further integrated into the analysis using an over-representation criterion (log2 combined score > 2), where the combined score represents a comprehensive measure of enrichment significance that incorporates both the *p*-value from Fisher’s exact test and the z-score for deviation from expected rank, thereby providing enhanced statistical power to identify biologically relevant pathways while controlling for both significance and effect size.

### 2.10. miRNA Target Prediction

Candidate miRNA-mRNA interactions were predicted using TargetScan 5.1, considering both conserved and non-conserved binding sites. Filtering was restricted to differentially expressed mRNA and miRNA identified by RNA-Seq, requiring an inverse correlation between mRNA and miRNA expression levels. Target similarity among differentially expressed genes was assessed using the Jaccard index. The resulting miRNA–mRNA interaction network was visualized using the locally installed Cytoscape v3.9.1 software, downloaded from https://cytoscape.org/ (accessed on 14 September 2025).

### 2.11. Construction of the ceRNA Network

A potential ceRNA network was constructed in two steps. First, mRNA-miRNA interactions were identified based on DEG. Second, mRNA-lncRNA and miRNA-lncRNA interactions were retrieved by integrating data from three databases: miRTarBase, lncRNA2Target, and lncbasev3. Before merging, filtering was applied to retain only differentially expressed mRNA, miRNA, and lncRNA, requiring an inverse correlation between lncRNA and miRNA expression levels and including all lncRNA-mRNA interactions. The final lncRNA-miRNA-mRNA ceRNA regulatory network was visualized using Cytoscape v.3.9.1.

### 2.12. lncRNA-mRNA Correlation

For the statistical analysis of lncRNA-mRNA cis-acting interactions, Pearson correlation analysis was performed. Correlation coefficients (r) were calculated for each lncRNA-mRNA pair across all samples (*n* = 16; 4 donors × 4 conditions) to quantify the strength and direction of their association. Corresponding *p*-values were computed to assess statistical significance, and the Benjamini–Hochberg procedure was applied to control the false discovery rate (FDR < 0.05) for multiple testing correction. Principal Component Analysis (PCA) was performed to assess overall variance and group separation. K-means clustering was applied to normalized expression data to explore global expression patterns among experimental conditions. The number of clusters was selected based on within-cluster sum of squares minimization and biological interpretability. Pearson correlation analysis was performed using normalized expression values to assess ncRNA–mRNA associations, excluding extreme outliers to reduce spurious correlations.

## 3. Results

### 3.1. Vitamin D-Mediated Regulation of Non-Coding RNA Networks in ZIKV-Infected Macrophages

#### 3.1.1. Vitamin D-Mediated Modulation of lncRNA Expression in ZIKV-Infected MDMs

**Figure 1:**
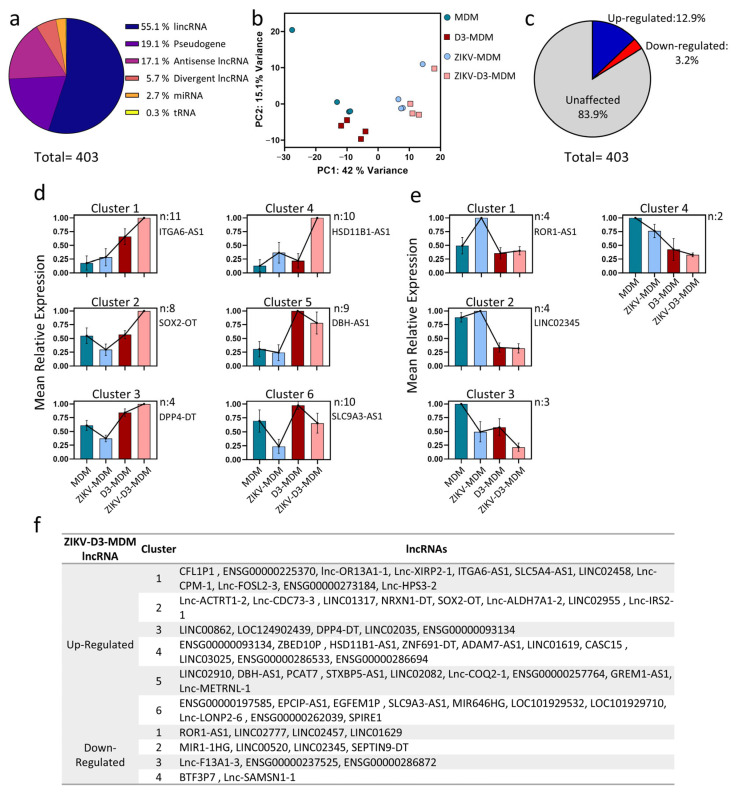
Alteration of lncRNA profiles by Vitamin D in Zika virus–infected MDMs. (**a**) Distribution of differentially expressed non-coding RNAs identified in ZIKV-D3-MDMs, categorized as lincRNAs, pseudogenes, antisense lncRNAs, divergent lncRNAs, miRNAs, and tRNAs. The total number of differentially expressed ncRNAs is indicated. (**b**) Principal component analysis (PCA) of differentially expressed lncRNAs across the four experimental conditions (MDM, D3-MDM, ZIKV-MDM, and ZIKV-D3-MDM). Each point represents a biological replicate, and the percentage of variance explained by each principal component is shown. (**c**) Pie chart illustrating the proportion of up-regulated, down-regulated, and unaffected lncRNAs in response to Vitamin D treatment in ZIKV-infected MDMs. (**d**) K-means clustering of up-regulated lncRNAs based on their normalized expression profiles across the four experimental groups. Each panel represents one cluster, and bars indicate mean relative expression values ± SEM. Representative lncRNAs for each cluster are indicated. (**e**) K-means clustering of down-regulated lncRNAs using the same analytical approach as in (**d**). (**f**) Summary table listing the up-regulated and down-regulated lncRNAs associated with each expression cluster identified by k-means clustering. The number of lncRNAs per cluster (n) is indicated.

Previously, we reported the efficacy of vitamin D in reducing ZIKV infection [9]. Here, we extended these findings by investigating the underlying regulatory mechanisms, with a particular focus on the role of lncRNAs as potential modulators of gene expression in ZIKV-infected macrophages differentiated in the presence or absence of VitD (ZIKV-D3-MDMs vs. ZIKV-MDM). To this end, we conducted a comprehensive analysis of lncRNA expression profiling using RNA-Seq. A total of 403 lncRNAs were found to be significantly altered across all the experimental groups (Table S1). These differentially expressed lncRNAs were further classified according to their genomic features, revealing the following distribution: 55.1% (*n* = 222) long intergenic non-coding RNAs (lincRNAs), 19.1% (*n* = 77) pseudogene-derived lncRNAs, 17.1% (*n* = 69) antisense lncRNAs, and 5.7% (*n* = 23) divergent lncRNAs (Figure 1a).

Next, we explore whether the expression profiles of the 403 lncRNAs could distinguish between the four experimental groups. Principal Component Analysis (PCA) reveals a clear separation between uninfected macrophages (MDMs and D3-MDMs) and their ZIKV-infected counterparts (ZIKV-MDM and ZIKV-D3-MDM) (Figure 1b). Furthermore, PCA shows that they exhibit specific, unique features despite the high similarity among ZIKV-infected macrophages. PCA also highlighted distinct transcriptional features, suggesting that VitD modulated the baseline expression of macrophages and shaped unique regulatory patterns upon viral infection. Consequently, we focused on lncRNAs exhibiting up- or down-regulation in response to VitD treatment during ZIKV infection. Out of the 403 lncRNAs analyzed, 83.9% (*n* = 338) displayed no significant changes in their expression levels when comparing ZIKV-D3-MDMs to ZIKV-MDMs. However, 12.9% (*n* = 52) were upregulated, while 3.2% (*n* = 13) displayed downregulation (Figure 1c and Table S2).

We applied the k-means clustering to categorize the expression profiles of 65 VitD-modulated lncRNAs (Figure 1d,f). For the upregulated lncRNAs, six distinct expression clusters were identified (Figure 1d). Cluster 1 comprises 11 VitD-dependent lncRNAs whose expression was further enhanced upon ZIKV infection (Figure 1d,f). Notably, this cluster contained ITGA-AS1, an lncRNA known to regulate ITGA6, a gene critical for cell adhesion and migration [18]. Cluster 2 encompasses 8 lncRNAs that show decreased expression levels following ZIKV infection (ZIKV-MDM) but increased expression in D3-MDM, with expression levels still notably stronger in ZIKV-D3-MDM (Figure 1d,f). Among these, SOX2-OT (SOX2 overlapping transcript) was of particular interest, as it overlaps with the SOX2 gene and plays a role in modulating its expression [19]. Furthermore, SOX2-OT is associated with vital cellular processes, including inflammation and oxidative stress [20].

Cluster 3 comprises four genes with an expression profile similar to Cluster 2, but distinguished by their basal up-regulation under VitD conditioning (Figure 1d,f). Within this cluster, DPP4-DT (dipeptidyl peptidase-4 distant transcript) was identified, which has been previously linked to ferroptosis [21]. Cluster 4 displays a particularly distinctive profile, as the lncRNAs are highly expressed in ZIKV-D3-MDM (Figure 1d,f). Among them, HSD11B1-AS1 has been associated with the modulation of inflammation [22]. Cluster 5 delineates a group of 9 lncRNAs specifically modulated in VitD-conditioned macrophages (D3-MDM), but whose expression decreases when these macrophages are subsequently infected with ZIKV (ZIKV-D3-MDM) (Figure 1d,f). Notably, DBH-AS1 was among these, an lncRNA reported as elevated in the peripheral blood of COVID-19-infected patients [23] and linked to the regulation of autophagy [24,25]. Finally, Cluster 6 consists of lncRNAs that are downregulated upon ZIKV infection in both MDM and D3-MDM. However, in VitD-conditioned (D3-MDM), their expression levels remain substantially higher compared to unconditioned (MDM) (Figure 1d,f). This cluster includes 9 lncRNAs, with SLC9A3-AS1 being particularly notable, as it has been described as a regulator of glycolysis [26].

Conversely, for the downregulated lncRNAs, we identified four distinct expression clusters (Figure 1e,f). Cluster 1 comprises lncRNAs expressed exclusively in infected MDMs (ZIKV-MDM), with no modulation observed under VitD conditioning (D3-MDM) or in infected macrophages (ZIKV-D3-MDM). This cluster contained 4 lncRNAs, among them ROR1-AS1, which has been previously described as an immunomodulator during SARS-CoV-2 infection [27]. Cluster 2, similar to Cluster 1, includes lncRNAs whose downregulation is exclusively driven by VitD, regardless of infection status (ZIKV-D3-MDM and D3-MDM, respectively) (Figure 1d,f). Notably, LINC02345 belongs to this group and has been associated with the regulation of copper-induced cell death [28]. Lastly, clusters 3 and 4 are characterized by decreased lncRNA expression in both VitD-conditioned (D3-MDM) and ZIKV-infected MDMs (ZIKV-D3-MDM), with three and two lncRNAs, respectively (Figure 1d,f). To date, however, no functional evidence has been reported for the lncRNAs in these two clusters.

#### 3.1.2. Vitamin D Modulates Cis-Acting lncRNAs to Regulate Gene Expression

One of the key molecular mechanisms by which lncRNAs exert their function is cis-acting regulation, whereby they physically link to specific genomic loci to control the expression of neighboring genes. A common strategy to infer the potential roles of lncRNAs in biological and pathological processes is co-expression analysis, which evaluates the correlation between lncRNA and mRNA expression profiles. To identify co-expressed lncRNA-coding gene pairs, we calculated Pearson correlation coefficients (PCC) based on the expression level of each differentially expressed lncRNA-mRNA pair, as previously [29]. This approach provides valuable insights into the mechanistic contribution of cis-acting lncRNAs during viral infection. In the case of ZIKV-D3-MDMs, our analysis focused on lncRNA-coding gene pairs with a PCC > 0.80 and a significance level of *p* < 0.05.

We identified a total of 129 lncRNA-mRNA interaction pairs, of which 124 exhibited positive correlations and 5 displayed negative regulations (Table S3). Since these interactions were located on the same chromosome, they are suggestive of a potential cis-acting regulatory relationship between the lncRNAs and their neighboring mRNAs.

To increase the specificity of our findings, we further refined the analysis by incorporating the genomic proximity between lncRNAs and coding genes. We specifically focused on interactions in which the distance between the lncRNA and its neighboring coding gene was less than 300 kilobases (Kb), either upstream or downstream. Applying this stringent criterion, we identified 15 lncRNA-coding gene pairs that fulfilled these conditions (Table S4). Notably, several interactions stood out, including SLC5A4-AS1-RFPL2 mRNA, PCAT7-FBP1 mRNA, DBH-AS1-SARDH mRNA, and LINC02345-SPRED1 mRNA. These lncRNAs have been previously implicated in the transcription [30], the modulation of viral pathogenesis [23], and the induction of endoplasmic reticulum stress [31].

**Figure 2:**
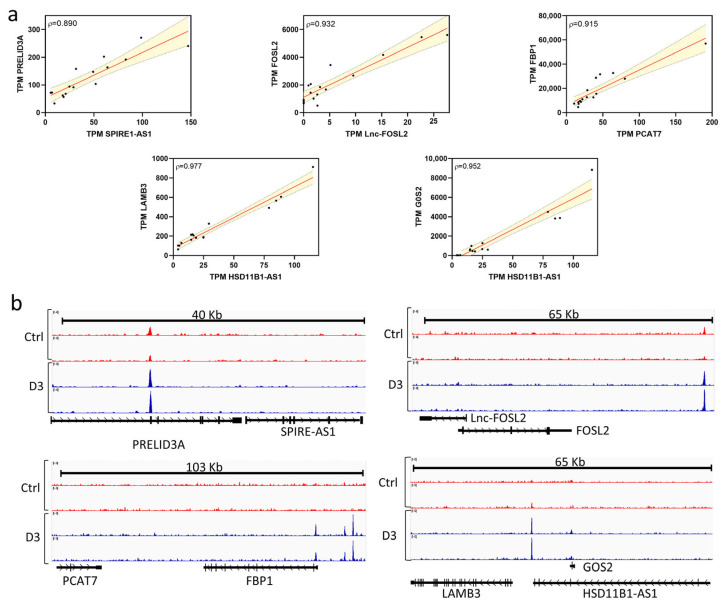
Vitamin D regulates cis-acting lncRNAs to modulate gene expression. (**a**) Pearson correlation analysis between selected cis-acting lncRNAs and their neighboring protein-coding genes. Each dot represents a paired expression value across samples. The solid red line indicates the linear regression fit, and the shaded area represents the 95% confidence interval. Pearson correlation coefficients (ρ) are shown within each panel. (**b**) Genomic tracks showing VDR ChIP-seq enrichment profiles in control macrophages (Ctrl, red) and vitamin D–treated macrophages (D3, blue). Tracks span genomic regions ranging from 40 to 103 kb, highlighting the proximity of VDR binding sites to the promoters of lncRNAs and their associated target genes. Gene annotations are shown below each track.

Next, we sought to determine whether VitD regulated these 15 lncRNA-mRNA pairs via the VDR signaling pathway. Our analysis revealed a statistically significant positive correlation between VitD exposure and the coordinated expression of specific lncRNA-mRNA pairs, including SPIR1-AS1-PRELID3A, lnc-FOSL2-FOSL2, PCAT7-FBP1, HSD11B1-AS1-LAMB3, and HSD11B1-AS1-G0S2, all of which are located within the same genomic loci (Figure 2a). Importantly, chromatin immunoprecipitation sequencing (ChIP-Seq) analysis demonstrated that VDR binding peaks are positioned within 50 kb of the transcription start site (TSS) of either the lncRNA or its paired coding gene. As shown in Figure 2b, genomic tracks of VDR ChIP-seq data illustrate enrichment profiles in THP-1 macrophages under control conditions (red) and following vitamin D treatment (blue), spanning up to 103 kb. The proximity of VDR binding sites to the promoters of both lncRNAs and their target genes strongly supports the notion that VitD/VDR signaling directly regulates the transcription of these cis-acting pairs. Taken together, these results indicate that VitD modulates cis-acting lncRNAs through VDR binding to regulatory regions of nearby genes, providing a mechanistic explanation for the coordinated upregulation observed in ZIKV-infected macrophages.

#### 3.1.3. Vitamin D Modulates miRNA Expression in ZIKV-Infected Macrophages

**Figure 3:**
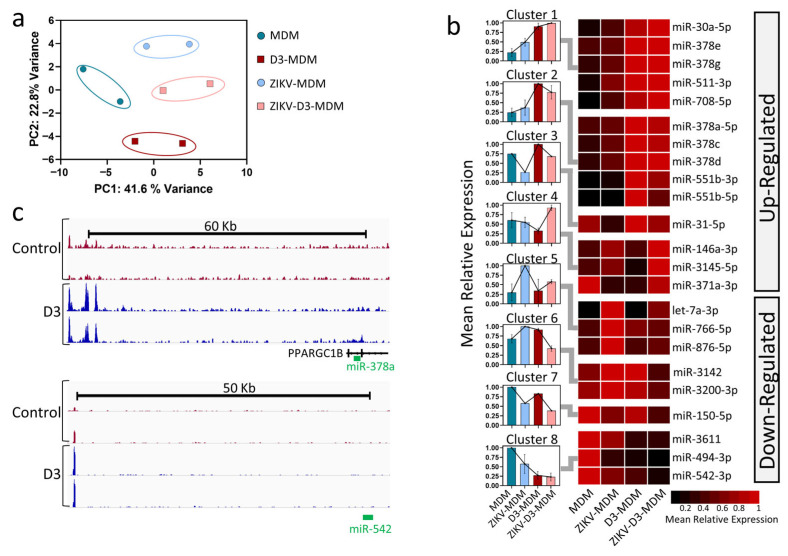
Vitamin D modulates miRNA expression in Zika virus–infected macrophages. (**a**) Principal component analysis (PCA) of miRNA expression profiles across four experimental conditions: uninfected macrophages (MDM), vitamin D–treated macrophages (D3-MDM), ZIKV-infected macrophages (ZIKV-MDM), and ZIKV-infected macrophages treated with vitamin D (ZIKV-D3-MDM). PC1 and PC2 indicate the percentage of total variance explained. Ellipses represent the 95% confidence interval for each group. (**b**) Heatmap of differentially expressed miRNAs grouped into eight clusters using k-means clustering based on their relative expression patterns across the four conditions. Mean normalized expression values are shown, with color intensity ranging from low (black) to high (red). Bar plots adjacent to each cluster represent the mean relative expression profile per condition. miRNAs are classified as upregulated or downregulated based on their expression trends in response to vitamin D and ZIKV infection. (**c**) Genomic tracks illustrating VDR ChIP-seq enrichment in control macrophages (red) and vitamin D–treated macrophages (blue). Tracks span genomic regions of approximately 50–60 kb surrounding the transcription start sites (TSS) of representative miRNAs (miR-378a and miR-542), highlighting potential VDR binding proximal to miRNA loci.

Next, we investigate whether the expression levels of miRNAs were altered in MDM, D3-MDM, ZIKV-MDM, and ZIKV-D3-MDM. Out of 2654 reported human mature miRNAs, 857 were detected across all four groups. PCA performed on these 857 miRNAs revealed a clear separation between the groups, indicating that their expression profiles can effectively discriminate each condition (Figure 3a). These findings suggest distinct sets of miRNAs are associated with the different experimental groups.

Building on our previous investigations into miRNAs modulated by VitD or ZIKV infection in macrophages [15,16,32], the present study specifically aimed to identify the expression pattern in VitD-conditioned and ZIKV-infected MDMs (ZIKV-D3-MDMs) compared to ZIKV-MDMs. We identified 23 differentially expressed (DE) miRNAs in ZIKV-D3-MDM (FDR ≤ 0.05 and |fold change| ≥ 1.5), of which 14 were up- and 9 were down-regulated (Figure 3b). To characterize these differences further, we applied a k-means clustering algorithm to the mean relative expression values of the 23 DE miRNAs. This analysis classified them into eight distinct clusters (clusters 1–8; Figure 3b) based on their dynamic expression profiles across the infected MDMs groups. Clusters 1–4 comprised miRNAs upregulated in response to VitD, whereas clusters 5–8 contained miRNA that were downregulated following VitD treatment (Figure 3b).

We next examined whether the differential expression of miRNAs was mediated through VDR. To address this, we analyzed publicly available VDR ChIP-seq data from THP1 macrophages treated with VitD for 24 h [33]. Our analysis revealed VDR binding peaks associated with the genomic loci of miR-378a-5p (cluster 2), located within the intronic region of the PPARGC1B gene, and miR-542-3p (Figure 3c). These findings indicated that, among the miRNAs differentially regulated in ZIKV-D3-MDMs, only miR-378a-5p and miR-542-3p appear to be direct transcriptional targets of VDR. This supports the notion that VDR binding events in proximity of the transcription start site of these miRNAs contribute to the modulation of their expression patterns in ZIKV-D3-MDMs.

#### 3.1.4. Vitamin D-Regulated miRNAs Modulate Key Biological Processes in ZIKV-Infected Macrophages

**Figure 4:**
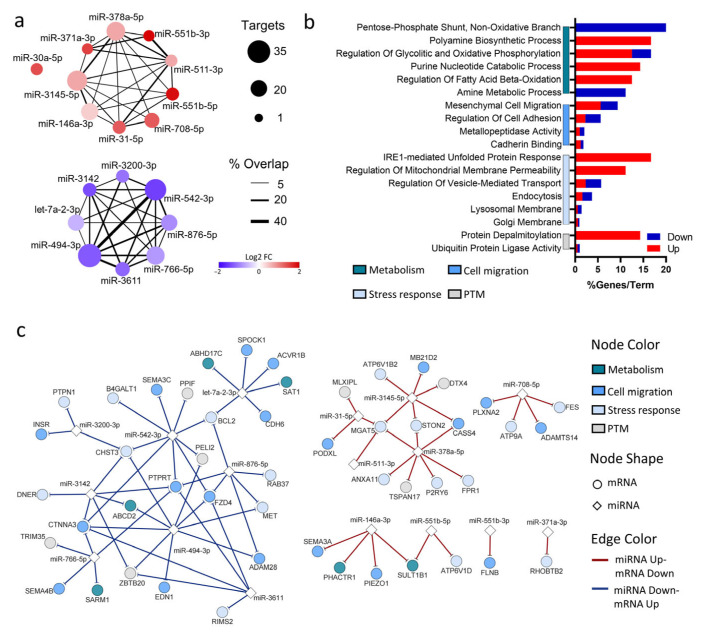
Vitamin D modulates miRNA–mRNA interaction networks in Zika virus–infected macrophages. (**a**) miRNA interaction network illustrating the potential regulatory impact of vitamin D–modulated miRNAs on their predicted mRNA targets. Upregulated miRNAs are shown as red nodes, while downregulated miRNAs are shown as blue nodes. Node size is proportional to the number of predicted target mRNAs per miRNA. Black edges represent shared target genes between miRNAs, with edge thickness indicating the percentage of target overlap. Node color intensity reflects the log2 fold change in miRNA expression. (**b**) Gene Ontology (GO) enrichment analysis of mRNAs predicted to be regulated by the differentially expressed miRNAs. Bars represent the percentage of genes associated with each enriched term. Red bars correspond to genes upregulated at the mRNA level, while blue bars indicate downregulated genes. Ontology terms are grouped into major functional categories, including metabolism, cell migration, stress response, and post-translational modification (PTM). (**c**) Integrated miRNA–mRNA interaction network showing predicted regulatory relationships between differentially expressed miRNAs and their target mRNAs. Node shapes indicate molecule type (diamonds: miRNAs; circles: mRNAs). Node colors denote functional classification of mRNAs, while edge colors indicate inverse expression relationships (red edges: miRNA upregulated/mRNA downregulated; blue edges: miRNA downregulated/mRNA upregulated).

To gain insights into the functional roles of the miRNAs modulated in ZIKV-D3-MDM, we examined the inverse relationship between miRNA expression and the expression of their predicted mRNA targets within the same samples. Predicted mRNA targets were identified for 17 of the 23 DE miRNAs, encompassing up- and down-regulated miRNAs (Figure 4a). This analysis uncovered 78 putative miRNA-mRNA interactions, underscoring the potential for target multiplicity in those 17 miRNAs. Furthermore, the up-regulated miRNA, miR-3145-5p, exhibited the highest number of predicted target sites (24) among mRNAs, while the down-regulated miRNA, miR-494-3p and miR-542-3p displayed the most significant number of predicted targets, with 31 and 27sites, respectively (Figure 4a).

Based on the integrative miRNA-mRNA analysis, we identified signaling pathways enriched among deregulated genes targeted by DE miRNAs (Figure 4b). Gene set enrichment analysis revealed that VitD-modulated miRNAs in ZIKV-infected MDMs predominantly regulate transcripts involved in cellular stress responses and metabolic processes. Specifically, we observed a downregulation of genes associated with the pentose phosphate pathway and, conversely, an upregulation of genes implicated in polyamine biosynthesis. Moreover, several genes that are linked to cellular stress pathways were upregulated, including those involved in the IRE1-mediated unfolded protein response (UPR) and the regulation of mitochondrial membrane permeability, suggesting that VitD may influence both metabolic reprogramming and stress adaptation during ZIFV infection.

Up-regulated miRNAs (miR-146a-3p, miR-551b-5p, miR-551b-3p, miR-371a-3p, miR-31-5p, miR-511-3p, miR-378a-5p, miR-3145-5p, and miR-708-5p) were predicted to mediate the downregulation of genes involved in cell migration (PLXNA2, CASS4, SEMA3A, PIEZO1, MB21D2, and PODXL), metabolism (PHACTR1 and SULT1B1), and stress response (FPR1, P2RY6, STON2 and FES) (Figure 4c). On the other hand, down-regulated miRNAs (miR-3142, miR-3200-3p, miR-542-3p, miR-876-5p, miR-766-5p, miR-3611, miR-494-3p, and let-7a-2-3p) were associated with the up-regulation of metabolism genes, including SAT1, ABCD2, SARM1, and ABHD17C (Figure 4c). Furthermore, several target genes of miR-494-3p were related to cell migration (FZD4, EDN1, ADAM28, and CTNNA3) (Figure 4c). In summary, these findings highlight an intricate regulatory network in which VitD-modulated miRNAs exert opposing effects on gene expression programs related to cell migration, metabolism pathways, and cellular stress response, underscoring their multifaceted roles in shaping macrophage responses to ZIKV under VitD conditioning.

#### 3.1.5. Regulation of Gene Expression by Vitamin D via Competing Endogenous RNA Mechanisms in ZIKV-Infected Macrophages: A Network Approach of lncRNA-miRNA-mRNA Interactions

**Figure 5:**
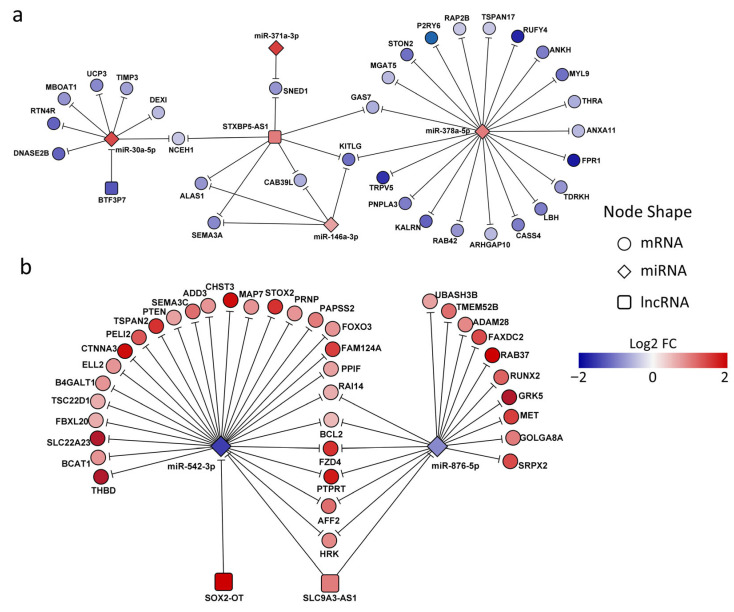
Vitamin D regulates gene expression through competing endogenous RNA (ceRNA) mechanisms in Zika virus–infected macrophages. Integrated ceRNA interaction networks illustrating potential cross-regulatory relationships among mRNAs, miRNAs, and lncRNAs. Networks are shown separately for (**a**) downregulated and (**b**) upregulated gene sets. Circular nodes represent mRNAs, diamond-shaped nodes represent miRNAs, and square nodes represent lncRNAs. Node color indicates the log2 fold change in expression, with blue representing downregulation and red representing upregulation. Edges denote predicted regulatory interactions based on shared miRNA targeting, highlighting putative ceRNA relationships.

To comprehensively understand posttranscriptional mRNA regulation, it is essential to recognize that miRNAs do not act in isolation; instead, their crosstalk with other non-coding RNAs (ncRNAs), such as lncRNAs, plays a pivotal role. To address this, we constructed a ceRNA network modulated in ZIKV-D3-MDM. Specifically, we examined the relationships between co-differentially expressed RNAs in VitD-conditioned and ZIKV-infected MDMs (ZIKV-D3-MDM vs. ZIKV-MDM). Using curated lncRNA-targets databases, we filtered potential interactions based on expression data to identify cases in which lncRNA-miRNA and lncRNA-mRNA interactions display opposite regulatory patterns. This analysis revealed that several downregulated mRNAs in ZIKV-D3-MDM were influenced by the lncRNA STXBP5-AS1, which cooperatively interacted with multiple miRNAs, including miRNA 378a-5p, miRNA 146-3p, miRNA 371a-3p, and miRNA 30a-5p (Figure 5a). Among these, miRNA 378a-5p emerged as the central node, showing the highest connectivity with 21 mRNA targets and the lncRNA, STXBP5-AS1, highlighting its prominent regulatory role within the ceRNA network. This interaction leads to down-regulation of GAS7, KITLG, CAB39L, SEMA3A, ALAS1, SNED1, and NCEH1. These finding suggests that lncRNA STXBP5-AS1 primarily exerts its effect through direct targeting of mRNAs rather than regulating miRNAs. The affected genes are functionally linked to cell growth (GAS7, KITIG, SEMA3A, and SNED1) and metabolism (ALAS1 and NCEH1). Furthermore, our analysis identified BTF3P7, a pseudogene that negatively regulated miR-30a-5p, supporting a potential sponge effect. In ZIKV-D3-MDM, the downregulation of BTF3P7 may relieve its inhibitory effect on miR-30a-5p, thereby enhancing miR-30a-5p activity and contributing to the downregulation of NCEH1, UCP3, and MBOAT1, all involved in the metabolism [34,35], as well as TIMP3, a regulator of extracellular matrix remodeling and macrophage proinflammatory response [36].

For the upregulated mRNA in ZIKV-D3-MDM, ceRNA network analysis identified miR-542-3p as the central hub, interacting with 27 mRNAs and two lncRNAs, SOX2-OT and SLC9A3-AS1 (Figure 5b). This network contributes to the upregulation of target genes involved in diverse biological processes. For instance, several miR-542-3p target genes are associated with cell proliferation, differentiation, and apoptosis, highlighting their role in maintaining cellular homeostasis. Notably, PTEN, FOXO3, and BCL2 regulate survival and apoptotic pathways, whereas CTNNA3 and FZD4 are involved in cell–cell adhesion and signaling cascades. Additionally, genes such as THBD and SEMA3C play a role in angiogenesis and vascular development. Beyond miR-542-3p interactions, our analysis also revealed that lncRNA SLC9A3-AS1 acts as a sponge for miR-876-5p, thereby promoting the upregulation of genes, including GRK5, RBA37, and PTPRT (Figure 5b), which are implicated in virus–host interactions and immune modulation. Collectively, these findings suggest that ceRNA-mediated regulation by SOX2-OT and SLC9A3-AS1 in ZIKV-D3-MDM involves miRNA sponging mechanisms that fine-tune the expression of genes governing cellular responses and virus–host dynamics during infection.

## 4. Discussion

ZIKV infection remains a major global health concern, underscoring the urgent need for an understanding of virus–host interactions to develop effective therapeutic strategies [37,38]. VitD has emerged as a promising immunomodulatory factor in several viral infections, including DENV, although the precise mechanisms highlighting its antiviral effects remain unclear [32,39]. Previous studies have shown that VitD modulates immune responses by influencing the expression of both miRNAs and mRNAs [15]. Moreover, lncRNAs have been identified as direct targets of VitD and may act as mediators of its signaling cascade [40]. Notably, both miRNAs and lncRNAs are key regulators of protein-coding genes [13]. Despite this evidence, the coordinate interplay among lncRNAs, miRNAs, and mRNAs in VitD-conditioned macrophages during ZIKV infection remains broadly understood.

This study investigates the molecular mechanisms underlying VitD-mediated immunomodulation during ZIKV infection, with particular focus on ncRNAs, specifically miRNAs and lncRNAs. Previous reports have shown that VitD can modulate the expression of specific lncRNAs [41]. By integrating RNA-seq and miRNA-seq data, we characterized the transcriptional landscape in VitD-conditioned macrophages after 24 h of ZIKV infection. Our analyses uncovered significant alterations in 65 lncRNAs and 23 miRNAs, with enrichment of pathways related to metabolism and immune responses. Given the critical role of ncRNAs in fine-tuning gene expression, these findings establish a novel regulatory framework that provides new insights into VitD–ZIKV interactions. Importantly, control macrophages were treated with 0.1% ethanol (EtOH), the vehicle for VitD, a concentration commonly used in in vitro studies and not reported to significantly alter ncRNA expression or viral replication in macrophages.

Notably, VitD-conditioned macrophages infected with ZIKV displayed distinct alterations in lncRNA expression, including the upregulation of lincRNAs, pseudogenes, antisense, and divergent transcripts. Among these, SOX2-OT, DPP4-DT, HSD11B1-AS1, DBH-AS1, and PCAT7 were significantly upregulated. Although these lncRNAs have not previously been associated with VitD, they have been linked to key biological processes such as cell adhesion, migration, inflammation, oxidative stress, ferroptosis, and autophagy. For instance, SOX2-OT has been shown to bind ILF3 and promote STAT3 phosphorylation, thereby activating TGF-β signaling [42]. DBH-AS1 regulates cell-cycle genes, enhancing proliferation and resistance to apoptosis [43], and has been linked to COVID-19 severity [23]. Interestingly, it is downregulated in hepatitis virus-associated hepatocellular carcinoma [44] but can be induced by HBx protein to promote proliferation through MAPK signaling [43]. Similarly, lncRNA *PCAT7* has been implicated in tumor progression via the ErbB/PI3K/Akt signaling pathway [45].

We also identified key lncRNAs involved in cis-regulatory mechanisms. For example, HSD11B1-AS1 regulates G0S2 in monocytes [22] and has been linked to protective roles in melanoma [46]. G0S2 is a critical regulator of lipid metabolism and inflammatory signaling, two processes that strongly influence flavivirus replication and host antiviral responses. In this context, the HSD11B1-AS1–G0S2 axis may represent a VitD-responsive regulatory mechanism linking macrophage immunometabolic reprogramming to antiviral defense during ZIKV infection. By modulating lipid utilization and inflammatory tone, this axis could indirectly affect membrane availability, cellular energy balance, and innate immune pathways required for efficient viral replication. Other antisense lncRNAs, such as Lnc-FOSL2-3 and SPIRE-AS1, may regulate nearby genes like FOSL2 and PRELID3A, respectively, both of which harbor VDR binding sites within 50 kb. FOSL2 is a regulator of antiviral cytokine responses [47] and has been reported to be upregulated in severe COVID-19 [48], underscoring its relevance in antiviral immunity. In contrast, PRELID3A plays a central role in mitochondrial lipid metabolism and the synthesis of cardiolipin (CL) [49]. Mitochondrial lipids, including CL, are critical regulators of immune function, and their depletion impairs cytokine production in macrophages [50]. Additionally, PRELID3A prevents apoptosis by facilitating phosphatidic acid (PA) transport within mitochondria under oxidative stress [51], maintaining mitochondrial membrane potential, enhancing respiratory chain function, and reducing reactive oxygen species production [52,53]. These functions suggest that PRELID3A could influence ZIKV pathogenesis in VitD-treated macrophages. Beyond antisense transcripts, other lncRNAs may exert regulatory effects on neighboring genes due to chromosomal proximity [54]. For instance, PCAT7 may regulate the adjacent gene FBP1, a critical enzyme in gluconeogenesis with established roles in macrophage polarization and immune responses [55].

Studies have shown that *FBP1* is activated during monocytic differentiation induced by 1,25-(OH)_2_D_3_, highlighting its responsiveness to VitD [56]. Moreover, FBP1 expression is directly regulated by VDR binding to its promoter [57,58]. During infection, IFN-γ enhances 1α-hydroxylase activity, leading to the conversion of VitD into its active form and, in turn, promoting FBP1 expression [59]. This interplay between VitD, IFN-γ, and FBP1 underscores the potential for PCAT7 and VitD to co-regulate immune responses during ZIKV infection. Nevertheless, experimental validation is still required to confirm PCAT7-mediated regulation of FBP1.

Collectively, these observations suggest that VitD-regulated lncRNAs may modulate ZIKV infection indirectly by shaping macrophage immunometabolic states. LncRNA-mediated regulation of genes involved in lipid metabolism, mitochondrial function, and inflammatory signaling may influence viral replication by affecting membrane composition, energy availability, and innate immune activation. In this context, regulatory axes such as HSD11B1-AS1–G0S2 and PCAT7–FBP1 may contribute to metabolic reprogramming that limits viral propagation while sustaining antiviral immune responses.

miRNA expression was also markedly altered by VitD in ZIKV-infected macrophages. Consistent with previous reports of VitD-mediated modulation of miRNAs in other arboviral infections [32,60,61]. Here, we identified several miRNAs involved in regulating metabolism, stress responses, and macrophage function [62,63,64]. Among the most notable, let-7a was significantly downregulated; this miRNA directly targets Tet2, a key regulator of IL-6 production and TCA cycle metabolites in macrophages [65,66]. Another downregulated miRNA, miR-494-3p, exhibits dynamic expression during macrophage polarization, being suppressed in M1 and induced in M2 phenotypes [67]. It also regulates components of the Wnt signaling pathway [68] and modulates C/EBP-β activation via Nrdp1 binding [67,69]. In contrast, among the upregulated miRNAs, miR-146a-3p emerged as a central regulator of the immune response. This miRNA suppresses pro-inflammatory and antiviral responses in microglial cells during ZIKV infection [70] by targeting TRAF6, a key adaptor protein in antiviral signaling [71]. Through the suppression of TRAF6, miR-146a-3p may attenuate excessive TLR-mediated inflammatory signaling, contributing to a balanced antiviral response that limits immunopathology while potentially restricting ZIKV replication. Additionally, upregulated miRNAs include miR-3145, which has been shown to inhibit influenza virus replication [72], as well as miR-708-5p and miR-378a-5p, both previously linked to immune regulation and viral infections [73,74].

We further identified several ceRNA networks that may underlie the observed changes in mRNA expression. Our findings are consistent with studies in other biological systems, highlighting the broad significance of ceRNA regulation across viral infections and VitD-mediated exposure [75,76,77]. Among these, STXBP5-AS1 emerged as a key ceRNA, supporting multiple miRNAs, and thereby promoting the downregulation of target mRNAs related to cell growth and metabolism. This observation aligns with findings in SARS-CoV-2-infected brain tissue, where STXBP5-AS1 functions as a ceRNA and potentially contributes to post-COVID neurological symptoms [78].

Additionally, lncRNAs such as SOX2-OT and SLC9A3-AS1 acted as sponges for miR-542-3p and miR-876-5p, respectively, leading to the upregulation of genes involved in metabolism and immune defense. This supports previous findings showing that SOX2-OT regulates inflammatory responses via miR-455-3p and miR-215-5p [79], and that SLC9A3-AS1 enhances gene expression by sequestering miRNAs such as miR-760 and miR-486-5p [80,81]. Furthermore, lncRNA STXBP5-AS1 has been reported to be upregulated by Rh2 via promoter hypomethylation and to function as a ceRNA by sequestering the oncogenic miR-4425 [82]. This lncRNA also promotes proliferation and invasion in cervical cancer cells by modulating the miR-96-5p/PTEN axis [83]. Altogether, these findings, along with our results, support a model in which ceRNA-mediated gene upregulation contributes to the molecular mechanisms underlying viral pathogenesis.

Interestingly, our study also identified BTF3P7, a pseudogene acting as a sponge for miR-30a-5p. Although research on pseudogenes as ceRNAs is still emerging, their regulatory potential is increasingly recognized. For instance, IFITM4P has been shown to function as a ceRNA for miR-24-3p in influenza A virus-infected cells, thereby modulating the expression of antiviral proteins IFITM1–3 [84]. Our results contribute to this growing body of evidence, suggesting that pseudogene-mediated ceRNA networks may play a role in shaping antiviral responses.

Beyond ZIKV infection, the regulatory interactions identified here are likely to be relevant in a broader viral and inflammatory context, as ncRNA-mediated regulation represents a conserved mechanism across diverse infectious diseases. Notably, VitD preferentially modulated the expression of specific lincRNAs, including SOX2-OT, DPP4-DT, HSD11B1-AS1, DBH-AS1, and PCAT7, which are implicated in metabolism, apoptosis, and cell proliferation. Although functional validation of apoptosis-related outcomes was beyond the scope of this study, the consistency of the transcriptomic signatures and their concordance with known biological pathways support the robustness of our conclusions and highlight the potential biological relevance of these ncRNA networks.

Together, these findings reveal a complex regulatory network of lncRNA–miRNA–mRNA interactions modulated by VitD in ZIKV-infected macrophages. This landscape uncovers previously unrecognized layers of post-transcriptional gene regulation during infection, suggesting potential molecular targets for therapeutic intervention. Nonetheless, our study is limited by the lack of functional validation, as the conclusions are primarily based on bioinformatics predictions and network-based analyses. Future work employing gene knockdown or overexpression approaches, luciferase reporter assays to validate predicted miRNA–lncRNA/mRNA interactions, RNA immunoprecipitation, and viral replication assays in genetically modified macrophages will be essential to confirm these predicted interactions and establish their biological relevance. Therefore, the present findings should be interpreted as hypothesis-generating, providing a foundation for subsequent experimental validation.

In summary, both miRNAs and lncRNAs are dynamically regulated by VitD during ZIKV infection, orchestrating immune and metabolic responses through diverse regulatory mechanisms. By integrating RNA-seq and miRNA-seq analyses, this study reveals previously unrecognized regulatory layers in VitD signaling in macrophages and identifies novel ncRNA candidates with potential as therapeutic targets in viral infections and immune-related disorders.

## 5. Conclusions

This study provides the first integrated transcriptomic analysis of lncRNA–miRNA–mRNA interactions in VitD-conditioned macrophages during ZIKV infection, revealing a complex regulatory landscape that coordinates immune and metabolic responses. We identified 65 differentially expressed lncRNAs and 23 miRNAs, including key VitD-responsive lncRNAs (SOX2-OT, HSD11B1-AS1, DPP4-DT, DBH-AS1, PCAT7) and miRNAs (miR-146a-3p, let-7a, miR-494-3p) that regulate critical processes such as inflammation, lipid metabolism, and macrophage polarization.

Our analysis uncovered putative regulatory axes (HSD11B1-AS1–G0S2, PCAT7–FBP1) linking VitD-driven immunometabolic reprogramming to antiviral defense, as well as extensive ceRNA networks in which lncRNAs sequester miRNAs to modulate genes involved in immune signaling and viral pathogenesis. These findings establish a novel framework for understanding VitD-mediated immunomodulation and identify promising ncRNA candidates for therapeutic targeting in arboviral infections.

While these results are primarily hypothesis-generating and require experimental validation through functional assays, they provide a comprehensive foundation for mechanistic studies and highlight the therapeutic potential of ncRNA-targeted interventions in viral infections and immune-related disorders.

## Data Availability

All relevant data are within the paper and its Supporting Information files. Raw sequencing data are available in the GEO database under accession number GSE209698.

## Supplementary Materials

The following supporting information can be downloaded at: https://www.mdpi.com/article/10.3390/pathophysiology33010015/s1, Supplementary Table S1: Differentially expressed lncRNAs across all pairwise comparisons; Supplementary Table S2: Differentially expressed lncRNAs in the ZIKV-D3-MDM group compared with ZIKV-MDM as the reference group; Supplementary Table S3: Cis acting lncRNAs pairs modulated in ZIKV-D3-MDM; Supplementary Table S4: Pairs of cis-acting lncRNAs modulated in ZIKV-D3-MDM within up to 500 kb.

## Abbreviations

The following abbreviations are used in this manuscript:

VitD	Vitamin D
ZIKV	Zika virus
MDMs	Monocyte-derived macrophages
lncRNAs	Long non-coding RNAs
miRNAs	MicroRNAs
ceRNA	Competing endogenous RNA
Kb	Kilobases
ChIP-Seq	Chromatin immunoprecipitation sequencing
UPR	Unfolded protein response
